# Prevalence of Poor Mental Health and Cognitive Status among Middle-Aged Adults and Its Predictors in Relation to Polyphenols Intake

**DOI:** 10.21315/mjms2019.26.3.6

**Published:** 2019-06-28

**Authors:** Hanisah Rosli, Suzana Shahar, Normah Che Din, Hasnah Haron, Nor Fadilah Rajab

**Affiliations:** 1 Community Rehabilitation and Ageing Research Centre (H-Care), Faculty of Allied Health Sciences, Universiti Kebangsaan Malaysia, Jalan Raja Muda Abdul Aziz, Kuala Lumpur, Malaysia; 2 Faculty of Allied Health Sciences, Cyberjaya University College of Medical Sciences, Persiaran Bestari, Cyberjaya, Selangor Darul Ehsan, Malaysia; 3 Dietetics Program, Faculty of Allied Health Sciences, Universiti Kebangsaan Malaysia, Jalan Raja Muda Abdul Aziz, Kuala Lumpur, Malaysia; 4 Health Psychology Program, Faculty of Allied Health Sciences, Universiti Kebangsaan Malaysia, Jalan Raja Muda Abdul Aziz, Kuala Lumpur, Malaysia; 5 Nutritional Science Program, Faculty of Allied Health Sciences, Universiti Kebangsaan Malaysia, Jalan Raja Muda Abdul Aziz, Kuala Lumpur, Malaysia; 6 Biomedical Science Program, Faculty of Allied Health Sciences, Universiti Kebangsaan Malaysia, Jalan Raja Muda Abdul Aziz, Kuala Lumpur, Malaysia

**Keywords:** mental health, cognitive, dietary factors, polyphenols, middle-aged adults

## Abstract

**Background:**

Decline in mental health and cognitive status starts to show its sign during middle-age and is affected by dietary factors, namely the polyphenols intake. Polyphenols have received attention in improving health issues related to aging, including decline in mental health and cognitive. The aim of this study is to determine the prevalence of poor mental health and cognitive status among middle-aged adults and its predictors in relation to polyphenols intake.

**Methods:**

Subjects’ food intakes were calculated by using dietary history questionnaire and food frequency questionnaire for polyphenols. The subjects’ mental health and cognitive status were measured by general health questionnaire-28 (GHQ-28) and Rey’s auditory verbal learning test (RAVLT).

**Results:**

More than 40% of middle-aged adults were identified as having signs of poor mental health. A total of 67.9% of the subjects had poor cognitive status according to RAVLT immediate recall. Hierarchical binary logistic regression indicated that fat intake was associated with somatic symptoms for both men [adjusted odds ratio (AOR) = 1.04; *P* < 0.05] and women (AOR = 1.06; *P* < 0.05). Intake of lignan (AOR = 1.071; *P* < 0.05) was associated with better RAVLT immediate recall among women. Additionally, high cholesterol (AOR = 3.14; *P* < 0.05) was associated with poor score of RAVLT delayed recall for women.

**Conclusions:**

Early detection of poor mental health and cognitive is crucial to prevent Alzheimer’s disease in old age.

## Introduction

With continuous increase in life expectancy due to the improvement in healthcare systems, the number of elderly population is expected to arise (1, 2). This situation comes with the debilitating effects of chronic pathologies as the population grows older. However, of all the changes which occur during the aging process, deterioration in mental health and cognitive is perhaps the single most disabling condition (3). It is also the most feared part of getting old. Some mental health issues, such as depression and insomnia are known to be common among the aging society (4). Some mental functions, i.e. verbal and numerical abilities and general knowledge seem robust to aging, however, certain capabilities like memory, executive functions, processing speed and reasoning showed deterioration as one ages (5, 6).

Many factors are found to be associated with poor mental health and cognitive status related to aging. This include the presence of chronic diseases, cigarette smoking and social, mental and physical inactivity. In contrast, dietary intake such as omega-3 fatty acids and vitamin B are found to be protective against the deterioration of mental health and cognitive status. Recently, a number of studies have strongly supported the role for polyphenols in the prevention of degenerative diseases, particularly neurodegenerative diseases including poor mental health and cognitive status (7, 8). Polyphenols are secondary metabolites of plants and are found naturally in fruits, vegetables, cereals, olive, dry legumes, chocolate and beverages, such as tea, coffee and wine (9). Increasing number of studies have described the roles of polyphenols, in particular flavonoids, in the prevention of human disease related to aging, such as cognitive impairment and Alzheimer’s disease through their antioxidant properties (10). In a study by Krikorian et al., a total of eight men and four women, with mean age of 78 ± 5 years were supplemented with either Concord grape juice (Welsh’s) or placebo for 6–9 mL/kg body weight divided over three servings for 12 weeks (11). The result showed that intake group had significantly better verbal memory acquisition as compared to placebo group at 12 weeks intervention period. No significant effect on delayed verbal memory and spatial memory were found. However, the studies are still scarce for conclusive evidence can be made. Moreover, these studies mainly aim the elderly groups although the signs and symptoms of poor mental health and cognitive status related to aging may start during the middle-age, and thus, early intervention can be started earlier. Therefore, this study aims to determine the prevalence of poor mental health and cognitive status among middle-aged adults and its predictors in relation to polyphenols intake.

## Subjects and Methods

This cross-sectional study involves a total of 339 middle-aged adults (aged 45–59 years), recruited from a low-cost housing flats in Kuala Lumpur. Sample size was calculated using a formula by (12), with the level of precision and confidence interval set at 5% and 95%, respectively. Additionally, the estimation of prevalence was 10% according to the prevalence of cognitive impairment in middle-aged adults reported in (13). From the calculation, the sample size was estimated to be 138.

Informed consent was obtained after providing written and verbal information to the subjects and subsequently, personal data and health profiles were collected from the subjects. Ethical approval was obtained from the Secretariat for Medical Research and Innovation, Universiti Kebangsaan Malaysia (NN-084-2013). Subjects were assessed for anthropometric, dietary intake, mental health and cognitive status.

### Anthropometric Measurements

Anthropometric profiles of the subjects such as standing height, weight and waist and circumferences were taken twice by a trained nutritionist and later the mean value was recorded. The standing height and weight values were used to calculate the body mass index (BMI) (14). Additionally, the subjects were classified as having increased risk of metabolic risk if their waist-hip ratio were ≥ 0.90 and ≥ 0.85 for men and women, respectively (15).

### Dietary Intake

The Malay version of dietary history questionnaire was used to assess the subjects’ dietary intake (16). It included the type of food, quantity and time of food intake throughout the day. Subsequently, the amount of energy and nutrients intake by the subjects was calculated using NutritionistPro (Axxya Systems Stafford, USA).

The food frequency questionnaire (FFQ) for polyphenols was utilised to analyse the subjects’ intakes of polyphenols. The intake of total polyphenols, phenolic acids, flavonoids, stillbenes and lignan was then calculated by referring to Phenol-explorer database.

### Mental Health

The general health questionnaire-28 (GHQ-28) consisted of 28 questions and subscales of somatic symptoms, anxiety and insomnia, social dysfunction and severe depression (17). For this study, the Malay version of GHQ-28 was used to assess the mental health status of the subjects (18). A study was conducted among healthy adults in Malaysia indicated that the sensitivity and specificity of GHQ was 87.5% and 80.6%, respectively, when validated against Beck Depression Inventory. Additionally, positive predictive value and area under ROC curve was 70% and 0.84, respectively. Cronbach’s alpha was 0.93, whilst Kappa coefficient was 0.64 (18). Any score exceeding the threshold of 4 was considered as having the risk of poor mental health status.

### Cognitive Status

In order to assess the subjects’ cognitive functions, the Malay version of Rey auditory verbal learning test (RAVLT) was used. It was proven useful in evaluating verbal learning and memory, including proactive inhibition, retroactive inhibition, retention, encoding versus retrieval, and subjective organisation (19).

The RAVLT consists of two different lists (A and B) of 15 concrete nouns. Subjects were asked to read list A five times (Recall 1 to Recall 5) at a rate of one item per second. Free verbal recall was tested immediately after each presentation. Then list B was presented followed by a free recall of list B. Thereafter, recall of list A (A6) was examined without prior presentation of list A. Subjects were classified as having poor cognitive status if their scores were lower than mean—1.5 standard deviation (SD) (20).

A study by Boone et al. (21) shows that the values of sensitivity and specificity of RAVLT were 73.8% and 90.0%, respectively, when validated against patients aged 16 to 64 years. In a study by Jamaluddin et al. (22), Malay version of RAVLT showed good validity (factor analysis 0.66 to 0.98) and test-retest reliability (Pearson’s correlation ranged from 0.24 to 0.84). Additionally, a study by Baitil Husna (23) indicated that the Cronbach’s alpha values for Malay version RAVLT was 0.74 and 0.84 for learning and memory sections, respectively.

### Statistical Analysis

All data were analysed using Statistical Package for Social Sciences (SPSS) version 21. Normality test Kolmogorov-Smirnov test was carried out before any statistical analysis. Socio-demography, social, health, anthropometric, biochemical, dietary and cognitive data was presented in mean and standard deviation. Categorical variables such as physical activity, living arrangements, years of education and smoking habit were presented as frequency and percentage. The association of nutrients and polyphenols with mental health and cognitive status was conducted using univariate Pearson’s correlation. Subsequently, associations between categorical data and mental health and cognitive status were tested using chi-square (χ^2^) test. Binary logistic regression was carried out to determine the predictors of poor mental health and cognitive status for both men and women.

## Results

A total of 349 middle-aged adults participated in this study, with 34.7% (*n* = 121) men and 65.3% (*n* = 228) women. The mean age for the male and female subjects were 53.7 ± 3.8 and 52.0 ± 4.3 years, respectively. Most of the subjects were married, had education until secondary level, retired, living with family members and socially inactive (Table 1). Additionally, most subjects did not engage in regular physical activities, did not smoke and drink alcohol and had no intake of nutritional supplements (Table 2).

Table 3 shows the energy and nutrients intakes by the subjects. Men (1933.3 ± 559.8 kcal) showed higher intakes of energy as compared to women (1586.3 ± 493.0 kcal) (*t* = 3.161; *P* < 0.01). Higher macronutrients intakes such as protein (*t* = 3.061; *P* < 0.01), fat (*t* = 2.857; *P* <0.01), carbohydrates (*t* = 2.458; *P* < 0.05) were also observed in men as compared to women.

With respect to micronutrient intake, men (1311.1 ± 778.6 mg) showed significantly higher intake of sodium as compared to women (966.8 ± 578.9 mg) (*t* = 2.522; *P* < 0.05). Similarly, men (9.6 ± 3.0 mg) had higher niacin intake as compared to women (7.6 ± 3.0 mg) (*t* = 3.146; *P* < 0.01).

**Figure 1:**
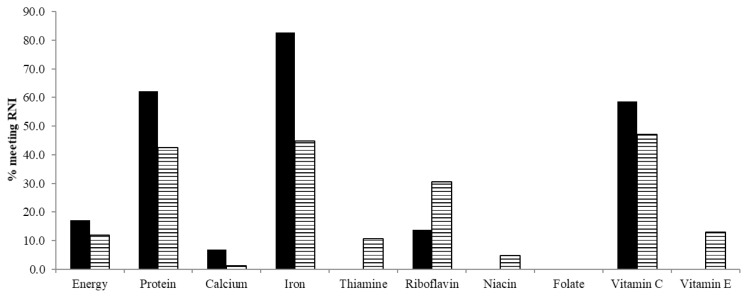
Percentage of subjects meeting RNI for energy and nutrients

As shown in Figure 1, more men met the Reference Nutrient Intake (RNI) requirements for energy, protein, calcium, iron and vitamin C as compared to women. In contrast, women showed higher intakes for thiamine, riboflavin, niacin and vitamin E. None of the subjects met the RNI requirement for folate.

As shown in Table 4, the total polyphenols intake were 1131.8 ± 568.8 mg/day and 947.2 ± 549.5 mg/day for men and women, respectively. Phenolic acid was the highest individual polyphenols group consumed by the subjects, with intakes of 452.7 ± 444.3 mg/day and 357.5 ± 393.0 mg/day for men and women, respectively. There was no significant difference in the total and individual classes of polyphenols intake between men and women.

**Figure 2:**
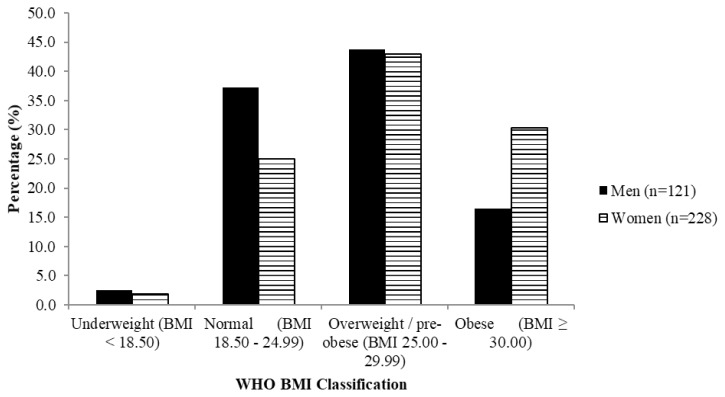
Classification of BMI

**Figure 3:**
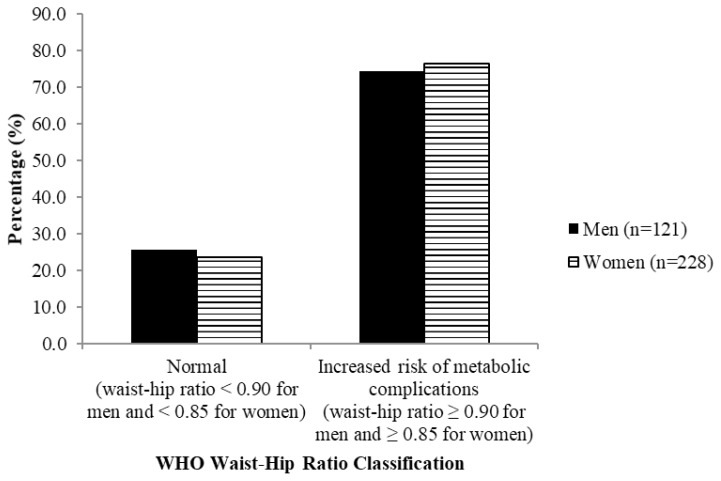
Classification of waist-hip ratio

Figures 2–3 show that most subjects were classified as overweight (about 45%) and had increased risk of metabolic complications (about 80%) according to waist-hip ratio, respectively.

**Figure 4:**
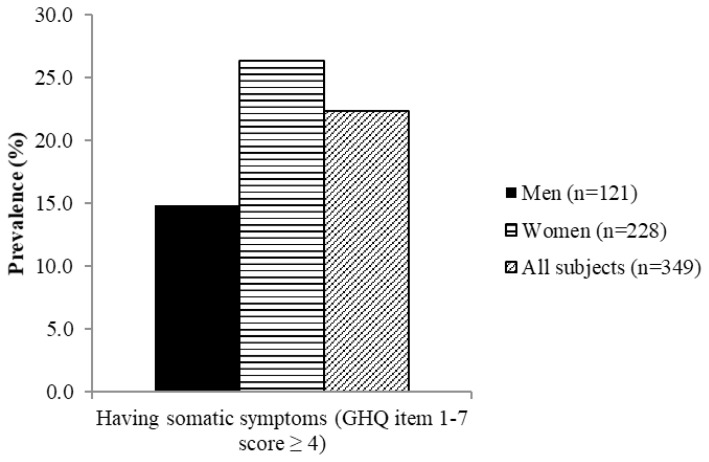
Percentage of subjects with somatic symptoms based on GHQ according to gender

**Figure 5:**
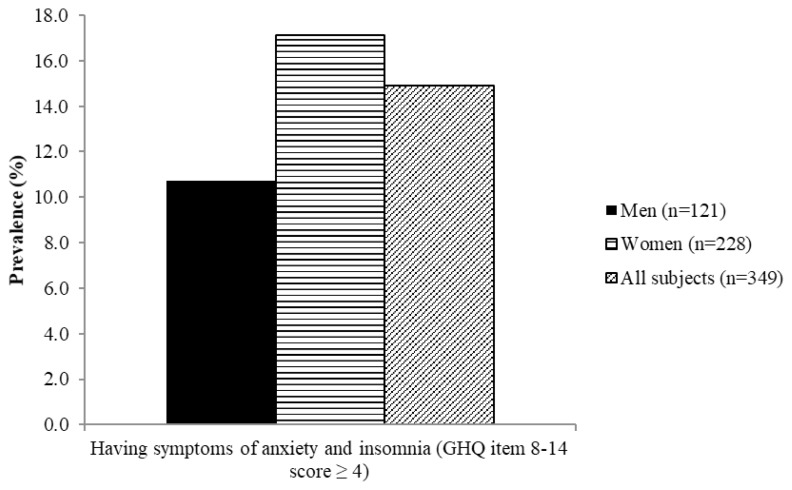
Percentage of subjects with symptoms of anxiety and insomnia based on GHQ according to gender

**Figure 6:**
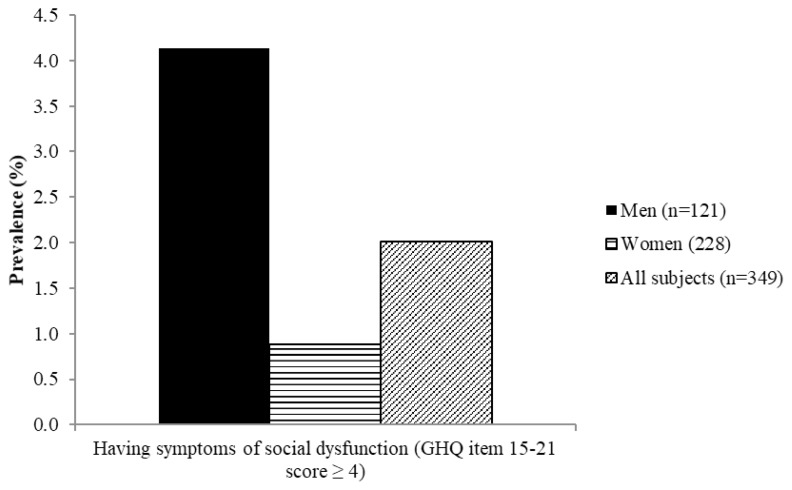
Percentage of subjects with symptoms of social dysfunction based on GHQ according to gender

**Figure 7:**
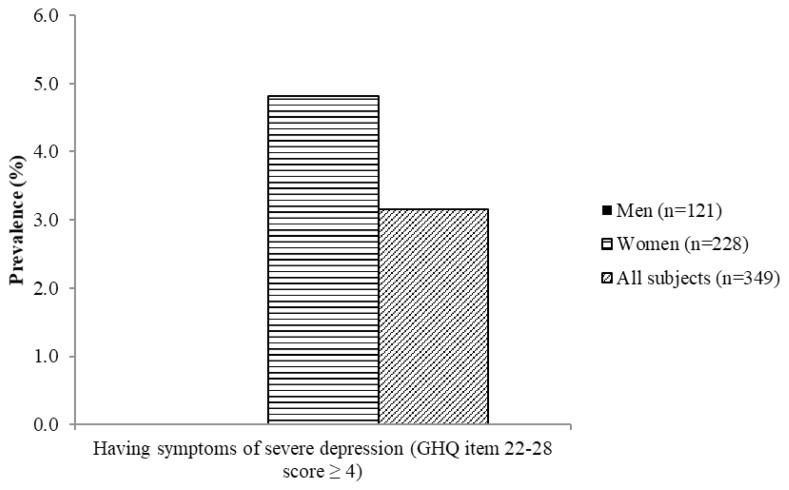
Percentage of subjects with symptoms of severe depression based on GHQ according to gender

**Figure 8:**
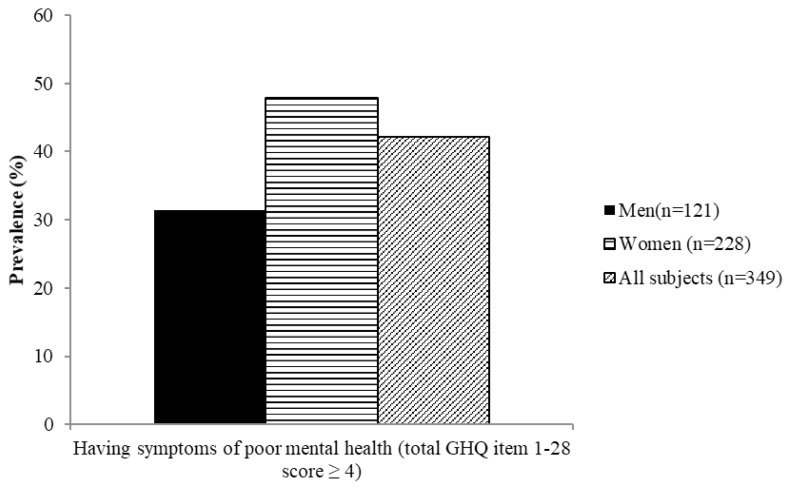
Percentage of subjects with symptoms of poor mental health based on overall GHQ score according to gender

Figures 4–8 show the prevalence of symptoms of poor mental health by gender disparities based on GHQ. About 26% of women were classified as having somatic symptoms as compared to men (14.9%). In addition, more than 17% of women had poor score in anxiety and insomnia category of GHQ as compared to men (10.7%). In contrast, social dysfunction was the only category with more men (4.1%) were found to have poor score as compared to women (0.9%). No men were found to show symptoms of severe depression as identified by GHQ, whereas 4.82% of women showed poor score in this category. Additionally, the total score of GHQ also showed that more women (47.81%) had poor score as compared to the male counterpart (31.4%).

**Figure 9:**
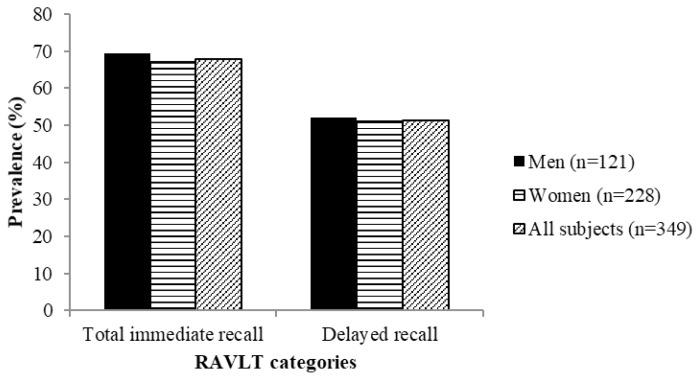
Percentage of subjects with symptoms of poor cognitive status based on RAVLT score according to gender

Figure 9 shows the prevalence of subjects with poor score in both RAVLT categories according to gender. More men were found to have poor score in both categories as compared to women. For total immediate recall, about 69.4% and 67.1% of men and women, respectively, were found to have poor score. Similarly, a total of 52.1% and 50.9% of men and women, respectively, had poor score in delayed recall categories.

Table 5 shows the association of nutrients and polyphenols intake with mental health and cognitive functions using univariate Pearson’s correlation. There was a significant correlation between phenolic acids and anxiety and insomnia (*r* = 0.227, *P* < 0.05), severe depression (*r* = 0.241, *P* < 0.05) and total GHQ score (*r* = 0.225, *P* < 0.05). In addition, there was a negative correlation between total flavonoids intake with somatic symptoms (*r* = −0.222, *P* < 0.05), anxiety and insomnia (*r* = −0.190, *P* < 0.05), total GHQ score (*r* = −0.231, *P* < 0.05) and RAVLT immediate recall (*r* = −0.213, *P* < 0.05). Other significant correlation include fat intake and somatic symptoms (*r* = 0.198, *P* < 0.05), calcium and anxiety and insomnia (*r* = −0.027, *P* < 0.05), iron and total GHQ score (*r* = −0.202, *P* < 0.05), vitamin A and RAVLT delayed recall (*r* = −0.206, *P* < 0.05), anthocyanins and total GHQ score (*r* = 0.344, *P* < 0.05) and lignan and RAVLT immediate recall (*r* = 0.263, *P* < 0.05).

Table 6 and Table 7 show the result of binary logistic regression analysis for the predictors of symptoms of poor mental health and cognitive in men and women, respectively, after controlling for education status, household income and energy intake. Analysis was not able to be carried out for social dysfunction and severe depression due to the low prevalence of both categories in this study. Demographic and health factors were analysed in Step 1 and Step 2, respectively. Step 3 was used to analyse dietary intakes, including polyphenols. The result shows that fat intake was found to be significantly associated somatic symptoms for both genders (*P* < 0.05). In addition, for women, iron intake was associated with total GHQ score (*P* < 0.05). For cognitive functions, lignan intake and high cholesterol were associated with RAVLT immediate and delayed recalls, respectively, among women (*P* < 0.05).

## Discussion

The number of middle-aged adults successfully recruited in this study is more than the sample size required based on the prevalence of cognitive impairment in middle-aged adults reported in 13. Nunley et al. (13). This study involved various low-cost housing flats located around Kuala Lumpur, and subjects were recruited through advertisement in their community.

This study shows that the intake of total polyphenols in the current study (994.6 ± 557.7 mg/day) was lower than those reported in other studies. It was reported that the intake of total polyphenols among subjects aged 60 years and above was 2770.7 ± 1552.4 mg/day (24). Additionally, a study reported that the intake of total polyphenols among 10,477 individuals aged 45 to 69 years of urban population in Poland was 1756.5 ± 695.8 mg/day (25). The study was conducted among the Western population, where fruit rich in polyphenols such as berries are highly accessible as compared in Malaysia. In addition, since the current study was conducted among individuals with low economic and education levels, the purchasing power of polyphenols-rich food could be hampered due to their background.

This study indicates that more women (26.3%) were identified as having somatic symptoms than men (14.9%). The situation is similar with another study where about 2.8% and 1.4% of women and men, respectively, were identified as having somatic symptoms (26). The much higher prevalence of symptoms of somatic symptoms in this study as compared to those reported in Silverstein (26) could be due to the different screening tools used in both studies. This study used GHQ as the screening tools and identified those with the symptoms, whilst the study by Silverstein (26) used data from National Comorbidity Survey involving adults from all age groups in the United States, with the clinical diagnosis of poor mental health.

The result shows that the prevalence of poor mental health (42.1%) according to total GHQ score is higher than those reported by several studies. The Malaysian National Health and Morbidity Survey (NHMS) reported that the prevalence of poor mental health for adults aged 45 to 59 years ranged from 24.9% to 27.7% (27), whilst a study conducted in Singapore had identified 16.6% of individuals with poor mental health according to total GHQ score (28). The high prevalence of poor mental health in this study could be due to most subjects in this study were recruited from low to middle income population. It was stated in NHMS that adults from low income families seems to be at risk of mental health problems (27). Individuals from socially and economically disadvantaged groups were more vulnerable to mental health problems (29).

In relation to cognitive status, the present study shows that women scored significantly higher than men in both immediate and delayed recalls of RAVLT. This is in contrast with the result by Lee at al. (30), where men showed better result in cognitive status as assessed by RAVLT scores. In another study by Teruya et al. (31), women scored better in the delayed recall category, but not in the immediate recall. In addition, a study by Sundermann et al. (32) showed that women outperformed men on verbal memory tasks throughout life. The gender difference in the cognitive functions may be related to the anatomical variations among both genders (32). There were significant gender and hippocampal volume/intracranial volume ratio (HpVR) interactions for immediate and delayed recall, determined using magnetic resonance imaging. It has been shown that the hippocampus regulates verbal memory (33). In addition, the volume of hippocampus is related to risk of amnestic mild cognitive impairment and Alzheimer’s disease (34). RAVLT is also a sensitive indicator of global cognitive functioning, not merely verbal learning skills (35).

This study shows that intake of total flavonoids was significantly associated with anxiety and insomnia. A study which was conducted among elderly people residing in old-folks homes indicated that those who had more than 50% of wastage of vegetables, a main source of total flavonoids, were 3.91 times higher to develop insomnia (36). Subjects with insomnia were 19.55 times more likely to develop depression. One of the explanations of the association of flavonoids intake with anxiety and insomnia is the affinity of flavonoids for central benzodiazepine receptors (37, 38). Benzodiapines are tranquilisers which have been used in the treatment of anxiety and insomnia. Flavonoids from the medicinal herb *Scutellaria baicalensis* Georgi, have been shown to manifest an affinity for the benzodiazepine receptor comparable to that of the synthetic anxiolytic diazepam (37). Both studies have further underlined the potential of flavonoids in improving the treatment of anxiety. The significant association of total flavonoids intake with better anxiety and insomnia status may shed some light on the roles of polyphenols in protecting mental health during old age. Due to this, more studies to identify the related metabolic pathways of polyphenols in aging brain should be conducted.

Analysis of hierarchical binary logistic regression with education status, household income and energy intake as the control variables was carried out to determine the predictors of poor mental health and cognitive status among the subjects. The result indicates that fat intake was a predictor of somatic symptoms for both men (AOR = 1.04) and women (AOR=1.06). Somatic symptoms such as dizziness, headaches, insomnia and fatigue, are related to other mental health factors, such as anxiety and depression (39). Another study also used GHQ to measure symptoms of mental health, Western pattern comprised diet such as meat pies, processed meats, pizza, chips, hamburgers, white bread, sugar, flavoured milk drinks and beer was associated with a higher GHQ score after adjustments for age, socioeconomic status, education, and health behaviours. In contrast, traditional dietary pattern comprised mainly vegetables, fruit, beef, lamb, fish and whole-grain foods was found to be protective against major depression and anxiety disorders with lower odds (40). Inflammatory and oxidative processes are thought to be responsible for the occurrence and maintenance of depressive disorders. Mediterranean diet which was high in vegetables, fruits, legumes, whole grains, fish, olive oil and low-fat dairy products, is negatively associated with inflammatory markers. In contrast, Western diets which is high in refined carbohydrates and fats is associated with higher levels of C-reactive protein, a marker of low-grade inflammation (40).

Additionally, this study found that iron intake is protective against poor mental health as assessed by total GHQ score among women (AOR = 0.689). A study was conducted among iron deficient and iron replete women aged 18 to 50 years to investigate the effects of iron supplementation and high iron diet for 12 weeks on several health parameters using SF-36 (41). Mental health and vitality scores were lower in iron deficient than iron replete women at baseline. Both scores of mental health and vitality showed improvements during the intervention phase, however the increase in serum ferritin was higher in the supplementation group as compared to high iron diet group. The mechanism on how iron affects mental health is still unknown, however, iron plays important roles in the functioning of several neurotransmitters, namely dopamine, serotonin and catecholamines (41).

In addition, the result of this study shows that the intake of lignan was associated with better RAVLT immediate recall score among women (AOR = 1.071). Similar result was also seen in a study higher dietary intake of lignan was found to be associated with better cognitive performance as assessed using mini mental state examination (MMSE) for post-menopausal women (42). Intake of lignan was measured using a validated FFQ and the analysis was adjusted for duration of fertile life, dietary intakes of saturated fat, monounsaturated fat, polyunsaturated fat, fatty fish, energy-adjusted fibre intake, energy-adjusted alcohol intake, and serum levels of estradiol. Women who were 20 to 30 years of post-menopause were the most prominent in the better cognitive performance. Similarly, in another study, women aged 60 to 75 years with high lignan intake was associated with better cognitive (43). Among the cognitive performance that were positively associated with high lignan intake was processing capacity and speed, and in executive function (43). Among the mechanisms proposed for this association is the attenuation of deposits of amyloid β-peptide (Aβ). These deposits may lead to the formation of neuritic plaques, and neurofibrillary tangles in vulnerable brain regions. These depositions are among the key features of Alzheimer’s disease (44).

Furthermore, this study shows that women with high cholesterol level were 3.14 times higher to have poor cognitive status as assessed by RAVLT delayed recall. The association of high cholesterol and cognitive was also studied by (45), where patients with and without familial hypercholecrolemia aged 50 years and above was recruited to determine the risk of mild cognitive impairment (MCI) in a population of patients with heterozygous familial hypercholesterolemia. Familial hypercholestemia is a condition involving LDL receptors dysfunction and is a life-long condition (45). There was a significant difference in the incidence of MCI between patients with (21.3%) and without (2.9%) familial hypercholesterolemia (*P* < 0.01). Furthermore, patients with familial hypercholesterolemia showed significantly worse performance in several cognitive measures such as mini-mental state examination and verbal learning, independent of apoE4 or apoE2 status. The findings suggested that early exposure to elevated cholesterol or LDL receptors dysfunction may be risk factors for mild cognitive impairment (45).

## Conclusion

This study indicated that more than 40% of middle-aged adults from low to middle socioeconomic status in Kuala Lumpur had poor mental health according to total GHQ score. Additionally, more than half of the adults recruited in this study had poor cognitive status according to RAVLT immediate recall score. Fat intake was found to be associated with somatic symptoms for both genders. For women, iron intake protective against poor mental health, presented by total GHQ score. Additionally, intake of lignan and high cholesterol were found to be associated with RAVLT immediate and delayed recall, respectively, for women.

The high prevalence of poor mental health and cognitive in this population requires immediate attention by health professionals and policy-makers for advanced research and remedial actions. By identifying the risk factors of these problems, various clinical trials can be conducted among the middle-aged adults to maintain good mental health and quality of life throughout old age. For upcoming research, recruitment of subjects should cover wider geographical area and ethnic groups to elucidate the real situation of mental health and cognitive status among middle-aged adults in Malaysia.

## Figures and Tables

**Table 1:** Subjects’ demographics, socioeconomic and social profiles according to gender [presented as number (%)]

	Men (*n* = 121)		Women (*n* = 228)		All (*n* = 349)	

	*N*	%	*N*	%	*N*	%
**Marital status**						
Single	8	6.6	7	3.1	15	4.3
Married	108	89.3	180	78.9	288	82.5
Divorced	3	2.5	13	5.7	16	4.6
Widowed	2	1.6	28	12.3	30	8.6
**Education status**						
No formal education	2	1.6	13	5.7	15	4.3
Primary education	18	14.9	63	27.6	81	23.2
Secondary education	83	68.6	150	65.8	233	66.8
Institute of higher learning	18	14.9	2	0.9	20	5.7
**Employment status**						
Not working	7	5.8	121	53.1	128	36.7
Working	86	71.1	17	7.4	103	29.5
Retired	28	23.1	90	39.5	118	33.8
**Living arrangements**						
Alone	5	4.0	4	1.8	9	2.6
With spouse	2	1.7	20	8.8	22	6.3
With children	2	1.7	39	17.1	41	11.8
With spouse and children	104	86.0	145	63.6	249	71.3
Others	8	6.6	20	8.7	28	8.0
**Participation in social activities**						
At least once a week	25	20.6	59	25.8	84	24.1
At least once a month	18	14.9	69	30.3	87	24.9
Less than once a month	18	14.9	41	18.0	59	16.9
No	60	49.6	59	25.9	119	34.1

**Table 2:** Subjects’ health behaviour according to gender [presented as number (%)]

	Men (*n* = 121)		Women (*n* = 228)		All (*n* = 349)	

	*N*	%	*N*	%	*N*	%
**Participation in physical activities**						
Everyday	14	11.6	24	10.5	38	10.9
3–5 times per week	8	6.6	11	4.8	19	5.4
1–2 times per week	27	22.3	35	15.4	62	17.8
No	72	59.5	158	69.30	230	65.9
**Smoking behaviour**						
Smoker	48	39.7	1	0.4	49	14.0
Former smoker	25	20.6	0	0.0	25	7.2
Non-smoker	48	39.7	227	99.6	275	78.8
**Intake of alcoholic beverages**						
Drinker	25	20.7	1	0.4	26	7.5
Former drinker	5	4.1	0	0.0	5	1.4
Non-drinker	91	75.1	227	99.6	318	91.1
**Intake of supplements**						
Fish oil	6	5.0	7	3.1	13	3.7
Multivitamins	5	4.1	22	9.6	27	7.7
Vitamin C	5	4.1	7	3.1	12	3.4
Calcium	3	2.5	9	4.0	12	3.4
Combination	3	2.5	22	9.6	25	7.1
Others	3	2.5	7	3.1	10	2.9
No	96	79.3	154	67.5	250	71.6

**Table 3:** Energy and nutrients intake according to gender (presented as mean ± SD)

Energy/Nutrients	Men (*n* = 121)	Women (*n* = 228)	*t*	95% CI	*P*
Energy (kcal)	1933.3 ± 559.8	1586.3 ± 493.0	3.161	129.5–564.6	0.002**
Protein (g)	71.6 ± 22.6	56.9 ± 22.2	3.061	5.2–24.2	0.003**
Fat (g)	66.2 ± 24.2	51.7 ± 23.4	2.857	4.45–24.57	0.005**
Carbohydrates (g)	262.6 ± 86.0	223.7 ± 68.9	2.458	7.54–70.24	0.016*
Calcium (mg)	402.4 ± 259.5	327.9 ± 230.9	1.454	−27.05–176.08	0.149
Iron (mg)	12.5 ± 6.0	12.1 ± 7.7	0.250	−2.72–3.51	0.803
Sodium (mg)	1311.1 ± 778.6	966.8 ± 578.9	2.522	73.85–614.79	0.013*
Potassium (mg)	1347.6 ± 412.3	1145.3 ± 500.1	1.962	−2.02–406.74	0.052
Vitamin A (μg RE)	1040.6 ± 699.7	799.4 ± 586.6	1.818	−21.69–503.97	0.072
Thiamine (mg)	0.6 ± 0.3	0.7 ± 0.3	−1.185	−0.21–0.05	0.239
Riboflavin (mg)	1.0 ± 0.5	0.9 ± 0.6	0.387	−0.18–0.27	0.700
Niacin (mg)	9.6 ± 3.0	7.6 ± 3.0	3.146	0.76–3.34	0.002**
Folate (μg)	51.2 ± 39.6	54.9 ± 44.7	−0.392	−22.17–14.85	0.696
Vitamin C (mg)	74.7 ± 48.8	115.6 ± 154.3	−1.400	−98.76–16.98	0.164
Vitamin E (mg)	3.4 ± 2.2	4.0 ± 2.5	−0.99	−1.57–0.522	0.324

* Significant at *P* < 0.05, using independent student’s *t*-test

** Significant at *P* < 0.00, using independent student’s *t*-test

SD = standard deviation; CI = confidence interval

**Table 4:** Subjects’ total and individual polyphenols intake (presented as mean ± SD)

	Men (*n* = 121)	Women (*n* = 228)	Total (*n* = 349)	*t*	*P*
**Phenolic acids (mg)**	452.7 ± 444.3	357.5 ± 393.0	382.9 ± 407.3	1.06	0.29
**Total flavonoids (mg)**	310.7 ± 228.5	249.8 ± 192.4	265.4 ± 203.0	1.37	0.17
**Anthocyanins (mg)**	3.77 ± 7.4	3.8 ± 7.3	3.8 ± 7.3	−0.01	0.99
**Stillbenes (mg)**	0.1 ± 0.0	0.1 ± 0.1	0.1 ± 0.1	0.11	0.92
**Lignan (mg)**	11.3 ± 15.4	8.0 ± 10.0	8.8 ± 11.6	1.18	0.24
**Total polyphenols (mg)**	1131.8 ± 568.8	947.2 ± 549.5	994.6 ± 557.7	1.52	0.13

No significant difference using independent student’s *t*-test

SD = standard deviation

**Table 5:** Association of nutrients and polyphenols intake with mental health and cognitive functions [presented as r]

	Somatic Symptoms	Anxiety & insomnia	Social dysfunction	Severe depression	Total GHQ	RAVLT immediate recall	RAVLT delayed recall
Energy (kcal)	−0.095	−0.010	0.005	−0.013	−0.051	0.165	−0.066
Protein (g)	−0.119	−0.18	−0.082	−0.070	−0.142	0.110	−0.065
Fat (g)	−0.198*	−0.105	−0.034	−0.048	−0.159	0.103	−0.093
Carbohydrates (g)	0.014	0.093	0.058	0.035	0.071	0.183	−0.027
Calcium (mg)	−0.109	−0.027*	−0.081	−0.023	−0.168	0.010	−0.067
Iron (mg)	−0.138	−0.190	−0.124	−0.089	−0.202*	0.071	−0.174
Sodium (mg)	0.008	−0.043	0.040	−0.021	−0.012	0.061	−0.130
Potassium (mg)	−0.164	−0.103	0.050	−0.062	−0.127	0.101	−0.071
Vitamin A (RE)	−0.093	−0.110	0.010	0.018	−0.084	−0.039	−0.206*
Thiamine (mg)	0.070	0.042	−0.058	−0.033	0.028	0.114	0.042
Riboflavin (mg)	−0.025	−0.124	−0.109	−0.049	−0.107	0.126	−0.001
Niacin (mg)	−0.174	−0.127	−0.062	−0.082	−0.174	0.177	−0.042
Folate (μg)	0.003	−0.048	−0.039	−0.053	−0.044	0.021	−0.139
Vitamin C (mg)	0.022	−0.088	0.102	−0.077	0.051	−0.003	−0.035
Vitamin E (mg)	0.021	−0.061	0.046	0.017	−0.002	0.015	−0.110
Phenolic acids (mg)	0.072	0.227*	0.079	0.241*	0.225*	0.099	−0.017
Total flavonoids mg)	−0.222*	−0.190*	−0.066	−0.105	−0.231*	−0.213*	−0.092
Anthocyanins (mg)	0.415	0.282	−0.008	0.067	0.344*	0.023	0.073
Stillbenes (mg)	0.655	0.295	0.343	0.052	0.482	−0.329	−0.004
Lignan (mg)	−0.086	0.023	−0.009	0.204	0.028	0.263*	0.056
Total polyphenols (mg)	−0.067	0.081	0.023	0.155	0.057	0.015	−0.062

* Significant at *P* < 0.05, using Pearson’s correlation

**Table 6:** Predictors of poor mental health and cognitive in men

	Variable	Adjusted odds ratio (95%CI)	*R^2^*	*P*
Somatic symptoms	Step 1		0.040	0.228
	Education status	0.536 (0.201–1.429)		0.213
	Household income	0.575 (0.218–1.516)		0.263
	Step 2		0.143	0.048*
	Energy	1.001 (0.999–1.003)		0.250
	Fat	1.041 (0.922–1.002)		0.048*
	Total flavonoids	0.998 (0.995–1.000)		0.078

* Significant at *P* < 0.05, using hierarchical binary logistic regression. The control variables were education status, household income and energy intake.

There are no significant predictors for (i) anxiety & insomnia, (ii) total GHQ, (iii) RAVLT immediate recall and (iv) RAVLT delayed recall

**Table 7:** Predictors of poor mental health and cognitive in women

	Variable	Adjusted odds ratio (95%CI)	*R^2^*	*P*
Somatic symptoms	Step 1		0.026	0.703
	Education status	0.562 (0.184–1.717)		0.311
	Household income	1.020 (0.333–3.127)		0.972
	Participation in social activities	0.769 (0.244–2.423)		0.654
	Step 2		0.157	0.052
	Energy	1.002 (0.999–1.004)		0.145
	Fat	1.062 (0.893–1.007)		0.026*
	Total flavonoids	0.999 (0.996–1.002)		0.483
Total GHQ	Step 1		0.047	0.524
	Education status	1.057 (0.134–8.344)		0.958
	Household income	1.298 (0.174–9.669)		0.799
	Step 2		0.474	0.013
	Energy	1.002 (0.999–1.004)		0.182
	Iron	0.689 (0.479–0.991)		0.045*
	Phenolic acids	0.999 (0.994–1.003)		0.504
	Total flavonoids	1.001 (0.997–1.005)		0.753
	Anthocyanins	1.392 (0.836–2.317)		0.203
RAVLT immediate recall	Step 1		0.130	0.040*
	Education status	0.219 (0.056–0.861)		0.030*
	Household income	2.230 (0.679–7.328)		0.186
	Step 2		0.249	0.090
	Energy	1.000 (0.999–1.002)		0.544
	Total flavonoids	1.000 (0.997–1.004)		0.808
	Lignan	1.071 (1.007–1.139)		0.028*
RAVLT delayed recall	Step 1		0.095	0.050
	Education status	0.322 (0.111–0.938)		0.038
	Household income	2.017 (0.731–5.565)		0.176
	Step 2 (against poor score)		0.159	0.038*
	High cholesterol	3.144 (1.032–9.615)		0.044*
	Step 3		0.172	0.641
	Energy	1.000 (0.998–1.001)		0.407
	Vitamin A	1.000 (0.999–1.001)		0.951

* Significant at *P* < 0.05, using hierarchical binary logistic regression. The control variables were education status, household income and energy intake.

There are no significant predictors for (i) anxiety insomnia and

## References

[1] Oeppen J, Vaupel JW (2002). Demography: broken limits to life expectancy. Science.

[2] Lutz W, Sanderson W, Scherbov S (2008). The coming acceleration of global population ageing. Nature.

[3] Singh-Manoux A, Kivimaki M, Glymour MM, Elbaz A, Berr C, Ebmeier KP (2012). Timing of onset of cognitive decline: results from Whitehall II prospective cohort study. BMJ.

[4] Buysse DJ (2004). Insomnia, depression and aging. Assessing sleep and mood interactions in older adults. Geriatrics.

[5] Hedden T, Gabrieli JDE (2004). Insights into the ageing brain: a view from cognitive neuroscience. Nat Rev Neurosci.

[6] Park DC, Reuter-Lorenz P (2009). The adaptive brain: aging and neurocognitive scaffolding. Annu Rev Psychol.

[7] Tsao R (2010). Chemistry and biochemistry of dietary polyphenols. Nutrients.

[8] Ma ZF, Zhang H (2017). Phytochemical constituents, health benefits, and industrial applications of grape seeds: a mini-review. Antioxidants.

[9] Pandey KB, Rizvi SI (2009). Current understanding of dietary polyphenols and their role in health and disease. Curr Nutr Food Sci.

[10] Spencer JPE, El Mohsen MMA, Minihane A-M, Mathers JC (2008). Biomarkers of the intake of dietary polyphenols: strengths, limitations and application in nutrition research. Br J Nutr.

[11] Krikorian R, Nash TA, Shidler MD, Shukitt-Hale B, Joseph JA (2010). Concord grape juice supplementation improves memory function in older adults with mild cognitive impairment. Br J Nutr.

[12] Daniel WW (1999). Biostatistics: a foundation for analysis in the health sciences.

[13] Nunley KA, Rosano C, Ryan CM, Jennings JR, Aizenstein HJ, Zgibor JC (2015). Clinically relevant cognitive impairment in middle-aged adults with childhood-onset type 1 diabetes. Diabetes Care.

[14] World Health Organization (2006). BMI classifications.

[15] World Health Organization (2011).

[16] Suzana S, Earland J, Suriah AR (2000). Validation of a dietary history questionnaire against a 7-D weighed record for estimating nutrient intake among rural elderly Malays. Malays J Nutr.

[17] Goldberg DP (1978). Manual of the general health questionnaire.

[18] Yusoff MSB (2010). The sensitivity, specificity and reliability of the Malay-version 12-items general health questionnaire-30 in detecting distressed medical students. EIMJ.

[19] Schoenberg MR, Dawson KA, Duff K, Patton D, Scott JG, Adams RL (2006). Test performance and classification statistics for the Rey auditory verbal learning test in selected clinical samples. Arch Clin Neuropsychol.

[20] Geffen G, Moar KJ, O’Hanlon AP, Clark CR, Geffen LB (1990). Performance measures of 16 to 86-year-old males and females on the auditory verbal learning test. Clin Neuropsychol.

[21] Boone KB, Lu P, Wen J (2005). Comparison of various RAVLT scores in the detection of noncredible memory performance. Arch Clin Neuropsychol.

[22] Jamaluddin R, Othman Z, Musa KI, Muhammad Alwi MN (2009). Validation of the Malay-version of auditory verbal learning test (MVaVLT) among schizophenia patients in HUSM, Malaysia. ASEAN J Psychiatr.

[23] Baitil Husna Z (2015). Aktiviti gaya hidup dan fungsi kognitif dalam kalangan warga emas yang tinggal dalam komuniti di Malaysia. Master diss.

[24] Suzana S, Chiah HL, Hasnah H (2014). Development and validation of food frequency questionnaire (FFQ) for estimation of the dietary polyphenol intake among elderly individuals in Klang Valley. Malays J Health Sci.

[25] Grosso G, Stepaniak U, Topor-Madry R, Szafraniec K, Pajak A (2014). Estimated dietary intake and major food sources of polyphenols in the Polish arm of the HAPIEE study. Nutrition.

[26] Silverstein B (2002). Gender differences in the prevalence of somatic versus pure depression: a replication. Am J Psychiatry.

[27] Institute for Public Health (2015). The national health and morbidity survey 2015.

[28] Fones CSL, Kua EH, Ng TP, Ko SM (1998). Studying the mental health of a nation: a preliminary report on a population survey in Singapore. Singapore Med J.

[29] Ahmad N, Yusoff FM, Ratnasingam S, Mohamed F, Nasir NH, Sallehuddin SM (2015). Trends and factors associated with mental health problems among children and adolescents in Malaysia. Int J Cult Ment Health.

[30] Lee LK, Suzana S, Chin A-V, Noor Aini MY, Nor Fadilah R, Safiyyah AA (2012). Prevalence of gender disparities and predictors affecting the occurrence of mild cognitive impairment (MCI). Arch Gerontol Geriatr.

[31] Teruya LC, Ortiz KZ, Minett TSC (2009). Performance of normal adults on Rey auditory learning test. Arq Neuropsiquiatr.

[32] Sundermann EE, Maki PM, Rubin LH, Lipton RB, Landau S, Biegon A (2016). Female advantage in verbal memory: evidence of sex-specific cognitive reserve. Neurology.

[33] Ystad MA, Lundervold AJ, Wehling E, Espeseth T, Rootwelt H, Westlye LT (2009). Hippocampal volumes are important predictors for memory function in elderly women. BMC Med Imaging.

[34] Kantarci K, Weigand SD, Przybelski SA, Preboske GM, Pankratz VS, Vemuri P (2013). MRI and MRS predictors of mild cognitive impairment in a population-based sample. Neurology.

[35] Callahan CD, Johnstone B (1994). The clinical utility of the Rey auditory-verbal learning test in medical rehabilitation. J Clin Psychol Med Settings.

[36] Suzana S, Junaidah H, Sundar VV, Kong AYW, Chin SP, Samsul Anuar S (2011). Determinants of depression and insomnia among institutionalized elderly people in Malaysia. Asian J Psychiatr.

[37] Medina JH, Viola H, Wolfman C, Marder M, Wasowski C, Calvo D (1998). Neuroactive flavonoids: new ligands for the benzodiazepine receptors. Phytomedicine.

[38] Huen MSY, Hui KM, Leung JWC, Sigel E, Baur R, Wong JTF (2003). Naturally occurring 2′-hydroxyl-substituted flavonoids as high-affinity benzodiazepine site ligands. Biochem Pharmacol.

[39] Bjelland I, Dahl AA, Haug TT, Neckelmann D (2002). The validity of the hospital anxiety and depression scale: an updated literature review. J Psychosom Res.

[40] Jacka FN, Pasco JA, Mykletun A, Williams LJ, Hodge AM, O’Reilly SL (2010). Association of western and traditional diets with depression and anxiety in women. Am J Psychiatry.

[41] Patterson AJ, Brown WJ, Roberts DCK (2001). Dietary and supplement treatment of iron deficiency results in improvements in general health and fatigue in Australian women of childbearing age. J Am Coll Nutr.

[42] Franco OH, Burger H, Lebrun CEI, Peeters PHM, Lamberts SWJ, Grobbee DE (2005). Higher dietary intake of lignans is associated with better cognitive performance in postmenopausal women. J Nutr.

[43] Kreijkamp-Kaspers S, Kok L, Grobbee DE, de Haan EHF, Aleman A, van der Schouw YT (2007). Dietary phytoestrogen intake and cognitive function in older women. J Gerontol.

[44] Jeong EJ, Lee HK, Lee KY, Jeon BJ, Kim DH, Park JH (2013). The effects of lignan-riched extract of Shisandrachinensis on amyloid-β-induced cognitive impairment and neurotoxicity in the cortex and hippocampus of mouse. J Ethnopharmacol.

[45] Zambon D, Quintana M, Mata P, Alonso R, Benavent J, Cruz-Sanchez F (2010). Higher incidence of mild cognitive impairment in familial hypercholesterolemia. Am J Med.

